# Feasibility of a clinically integrated rehabilitation therapist in a Neuro-Oncology clinic

**DOI:** 10.1093/noajnl/vdad098

**Published:** 2023-08-16

**Authors:** Nicole L Stout, Jacob Greenfield, Sonikpreet Aulakh

**Affiliations:** Division of Hematology/Oncology, West Virginia University Cancer Institute, Morgantown, West Virginia, USA; Department of Health Policy, Management, and Leadership, West Virginia University School of Public Health, Morgantown, West Virginia, USA; School of Occupational Therapy, West Virginia University School of Medicine, Human Performance, Morgantown, West Virginia; Division of Hematology/Oncology, West Virginia University Cancer Institute, Morgantown, West Virginia, USA

**Most brain tumor patients experience morbidity in functional domains including physical, cognitive, and psychosocial.^1^ Therefore, rehabilitation is often recommended to optimize the activities of daily living. The underutilization of rehabilitation in current oncology care models is a challenge. Less than 9% of individuals with measurable functional morbidity receive rehabilitation referrals.^2,3^ This gap is magnified in rural areas where local supportive care is not as widely available.**

At the West Virginia University in the brain tumor clinic, an occupational therapist (OT) was embedded to evaluate functionality. WV is a rural state where patients drive approximately 50 minutes, one-way, to receive cancer care. Our goal is to reduce barriers to access rehabilitation services and minimize additional follow-up appointments.

Clinically integrated rehabilitation professionals in the cancer center could alleviate gaps in care and reduce the time to treatment for functional impairment.^4,5^ A prospective surveillance approach promotes early identification and management of functional morbidity.^6,7^ The purpose of this manuscript is to share the feasibility of an OT in the brain tumor clinic.

In March 2022, an onsite OT evaluated functional decline as determined by the NeuroOncologist. On an average, the clinic has 18–20 patients/week. The clinical workflow started with the NeuroOncologist conducting a brief functional screen based on current or changing performance status and determining if an OT assessment was needed.

**Figure 1.**
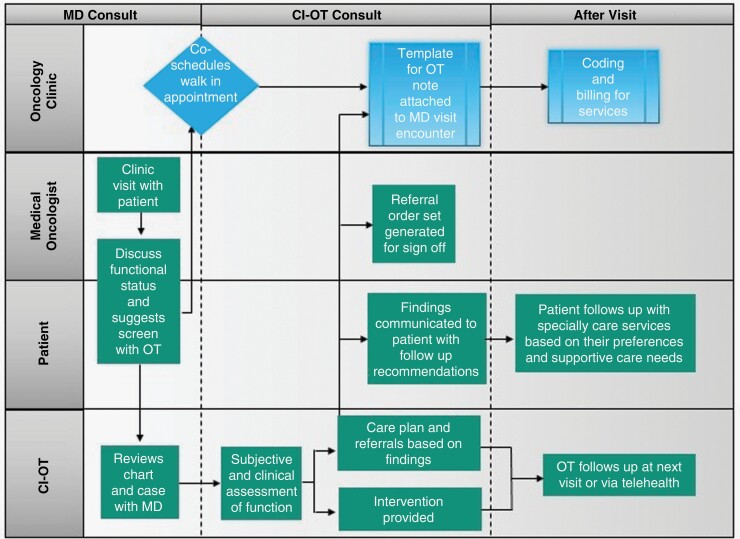
Clinically integrated occupational therapist workflow. Abbreviations: MD, physician; OT, occupational therapist; CI-OT, clinical integrated occupational therapist.

Several strategies including development of Electronic Health Records (EHR)to facilitate scheduling, documentation, and billing were undertaken to support the implementation of the OT. Walk-in appointments for the same-day OT enabled the flexibility in the visit. Clinic rooming procedures were modified to accommodate OT while not hampering the NeuroOncologist’s schedule (Figure 1).

We selected 3 areas of focus; *demand* for the OT assessment, *practicality* of the integrated service, and provide limited *preliminary evidence of efficacy*.^8^ We also estimated downstream utilization based on the referrals. We calculated *demand* by reviewing the overall utilization of the OT and compared this to overall clinic volume to determine a rate, with the assumption that this is also an indication of the prevalence of functional needs. We measured overall *practicality* by assessing the time, space, and workflow allocation. By tracking the OT recommendations, we evaluated the *efficacy* by identifying patients who need an intense supportive intervention.

Between March 2022 and August 2022, the OT attended 56.5% (13/23) of scheduled brain tumor clinics. During these clinics, the NeuroOncologist saw 316 patients and the OT assessed 61 (19.3%) patients for functional morbidity. The OT-generated referrals to home health services *n* = 32 (52.5%), Speech/audiology *n* = 10 (16.4%), physical therapy *n* = 9 (14.7%), OT *n* = 6 (9.8%), NeuroOpthalmology *n* = 3 (4.9%), community-based services *n* = 3 (4.9%) and social work *n* = 1 (1.6%).

Within the Neuro-Oncology clinic, 19.3% of patients were deemed appropriate for an OT. 10 (16.4%) of these individuals had functional needs that could be addressed with a low-intensity education and home program intervention. The OT provided this intervention with an intended follow-up.

The OT covered 13 out of 23 available brain tumor clinics over 6 months during which time 61 patients were assessed. The duration of time spent for the OT assessment ranged from 8-20 minutes (14 minutes average) for an estimated 88 patient care hours. The OT averaged seeing 4.7 patients/clinic covered and generated 4.5 referrals/clinic. 51 patients received appropriate referrals and 10 patients received point-of-care interventions.

Utilization estimates suggest that an OT could see up to 15 patients in a 5-hour medical clinic. This could result in an estimated 8–10 referrals to revenue-generating services within the health system.

This pilot care delivery model suggests that by screening functional needs of the brain tumor patients, a Neuro-Oncologist can utilize an on-site OT for a comprehensive evaluation. The current rate of 19.3% of patients referred for OT consult, while low compared to estimated morbidity burden in oncology patients,^9^ demonstrates improvement in referral trends. This clinically integrated approach facilitates engagement to better manage cancer treatment-related symptoms and morbidity.^5,10^

As reflected by the breadth of service referrals generated by the OT, this model enabled identification and management of functional deficits across physical, cognitive, psychological, and social domains at the point of care. A recent report from the National Cancer Institute-CONNECT (Comprehensive Oncology Network Evaluating Rare CNS Tumors) identified that psychosocial support needs, adapting to new limitations, and challenges with clinical care are the top issues brain cancer patients face. Neuro-Oncology navigators lack the specific skills and abilities to assess the individual across these domains of function, as a rehabilitation provider can.^4^ Having the OT to provide advice on interventions for self-care management obviates the need for additional visits for patients, especially in rural areas. This approach addresses access to care, heightens awareness of the patient’s functional needs, and equips patients with strategies to optimize function.

The screening approach used to identify patients for OT consultation was ad hoc and relied on the physician’s experience rather than a validated screening or assessment tool. A validated patient-reported outcome measure, such as the Patient-Reported Outcomes Measurement Information System (PROMIS) Cancer Function Brief 3D has better predictive validity, and could improve the sensitivity in identifying patients who are at risk for functional decline.

While the face-to-face time between the OT and patient was minimal, the overall clinic time should be considered when implementing this approach. The additional time in the exam room and additional staff time to accommodate this change were factors that impacted the overall clinic workflow and should be anticipated and accounted for.

We did not conduct a cost analysis of potential billable revenue nor of downstream revenue projections from the OT visits and referrals. These projection models should be considered in future work to demonstrate the potential for revenue generation from this consultative service.

While there is movement in the field of Neuro-Oncology to implement valid Patient-Reported Outcomes, there is an equivalent need to implement a care delivery model that can leverage PRO findings to manage symptom burden and reduce functional morbidity rather than just characterize patient functional morbidity.

In conclusion, this feasibility pilot project demonstrates that a clinically integrated rehabilitation professional adds value to the Neuro-Oncology team by improving detection and management of functional morbidity for individuals with brain tumors. Further exploration of this model is warranted to streamline supportive care services.
